# Early Symptom Warnings and Long-Term Health Conditions in Childhood Cancer Survivors: Insights from the Childhood Cancer Survivor Study and St. Jude Lifetime Cohort Study

**DOI:** 10.21203/rs.3.rs-7229897/v1

**Published:** 2025-09-15

**Authors:** I-Chan Huang, Madeline Horan, Wei Liu, Deo Srivastava, Matthew Ehrhardt, Daniel Mulrooney, Wassim Chemaitilly, Kirsten Ness, Justin Baker, Gregory Armstrong, Melissa Hudson, Kevin Krull

**Affiliations:** St. Jude Children's Research Hospital; Wake Forest University School of Medicine; PHASTAR; St. Jude Children's Research Hospital; St. Jude Children's Research Hospital; St. Jude Children's Research Hospital; University of Pittsburgh Medical Center; St. Jude Children's Research Hospital; Stanford University School of Medicine; St. Jude Children's Research Hospital; St. Jude Children's Research Hospital; Department of Epidemiology and Cancer Control, St. Jude Children's Research Hospital, Memphis

**Keywords:** Childhood Cancer, Chronic Health Conditions, Late Effects, Patient-Reported Outcomes, Survivors, Symptom Clusters

## Abstract

Symptom patterns in adult survivors of childhood cancer may signal risk for health deterioration and offer a foundation for risk stratification. 735 survivors completed sequential symptom surveys (T1, T2, T3) and clinical assessment for chronic health conditions (CHCs). Survivors were classified into four clusters: 1) low physical and emotional symptoms, 2) moderate physical and low emotional symptoms, 3) moderate physical and emotional symptoms, and 4) high physical and emotional symptoms. Survivors in cluster 4 vs. cluster 1 had an elevated risk of progressive total CHC burden and vascular, respiratory, neurologic, and musculoskeletal conditions (relative risk [RR] range: 1.24–2.53). Increased/persistently high symptom burden between T1-T2 increased risk for progressive total CHC burden, respiratory, and neurologic conditions (RR range: 1.30–2.23). Increased/persistently high T2-T3 symptom burden showed similar associations (all p’s < 0.05). This proof-of-concept study provides an empirical basis for developing and validating symptom-based prediction models and warning systems to support proactive survivorship care.

## INTRODUCTION

Childhood cancer and its treatment have been associated with a wide range of late effects,^1,2^ which can adversely impact physical and psychosocial function^3,4^ and contribute to premature mortality.^5^ The St. Jude Lifetime Cohort Study (SJLIFE) found that by age 50 years, adult survivors of childhood cancer experienced an average of 17 chronic health conditions (CHCs), of which 4.7 conditions were severe/disabling, life-threatening, or fatal, based on grades 3–5 of the Common Terminology Criteria for Adverse Events (CTCAE).^6^

Screening for and preventing the onset or worsening of CHCs is a primary task of cancer survivorship care. Currently, screening guidelines for childhood cancer survivors largely rely on clinical factors (e.g., cancer diagnosis, treatment history) to address the risk of late effects and inform subsequent follow-up care.^1,2,7,8^ This approach is challenging because survivors and their primary care providers may lack detailed knowledge of the survivor’s diagnosis, treatment, and associated risk of late effects.^9^ Moreover, this strategy does not consider socio-demographics, lifestyle, or perceived symptoms that may mediate or modify the relationships between cancer treatment and long-term health outcomes.^10^

Childhood cancer survivors experience symptoms across multiple domains, including cardiac, pulmonary, and sensory dysfunction, pain, fatigue, and poor memory and attention. Notably, 75% of childhood cancer survivors report multiple co-occurring symptoms,^11^ and a greater symptom burden is linked to poorer quality of life (QOL).^11^ When symptoms present together, their combined effect may exacerbate QOL impairments,^12^ and commonly co-occurring symptoms may reflect shared underlying mechanisms that contribute to greater disease severity.^13^ While prior studies have evaluated symptom clusters (defined as patterns of multiple, co-occurring symptoms) in pediatric cancer patients,^14^ few have focused on adult survivors of childhood cancer.^12^ Furthermore, existing studies are limited by small sample sizes (< 150 survivors) and a cross-sectional design. Expanding this line of research is crucial to guide the development of interventions aimed at reducing the health burden and QOL impairment among survivors with persistent symptom clusters.

Patient-reported symptoms, representing the manifestation of health abnormalities, can prompt medical consultation in individuals with and without a cancer history.^15^ In adult-onset cancer, symptom reporting during and after therapy has demonstrated independent prognostic value for survival, beyond socio-demographic and clinical factors.^16^ Collecting symptom data from this population has been shown to enhance patient-doctor communication, improve QOL, reduce emergency visits, and increase survival.^17^ While prognostic models for conditions, such as heart failure, ischemic heart disease/stroke, and hypertension/diabetes, have been developed in pediatric cancer survivors,^18^ systematic symptom assessment using standard tools remains underutilized in survivorship care.

It is clinically intuitive that symptoms often precede the diagnosis of CHCs and prompt medical attention, yet empirical evidence linking *early symptom clusters* to subsequent CHC development remains limited. Leveraging comprehensive longitudinal data from two well-characterized survivor cohorts, the SJLIFE and the Childhood Cancer Survivor Study (CCSS), this study examined symptom clusters and their changes over a 25-year period among adult survivors of childhood cancer, in relation to the progression of clinically ascertained CHCs, including new onset, persistent, and worsening conditions, while accounting for treatment history, socio-demographic characteristics, and lifestyle factors.

## METHODS

### Study Participants

Study participants were adult survivors of childhood cancer from both SJLIFE and CCSS, two retrospective cohort studies with prospective follow-up to characterize the etiology and late effects of childhood cancer. As of December 2020, SJLIFE included over 6,000 survivors of pediatric malignancies diagnosed at St. Jude Children’s Research Hospital (SJCRH) between 1962 and 2012, who returned periodically for clinical assessments.^19^ CCSS comprises 25,665 survivors diagnosed and treated at one of 31 institutions in North America, including SJCRH, between 1970 and 1999, and followed through periodic surveys.^5^ Approximately 40% of SJLIFE participants also participated in CCSS.

Eligible participants were survivors who 1) were aged ≥ 18 years; 2) completed at least three patient-reported outcome (PRO) surveys, including CCSS baseline (T1) and two follow-up (T2, T3) surveys via CCSS and/or SJLIFE; and 3) underwent a SJLIFE clinical assessment after T3. Survivors were excluded if PROs were proxy-reported, unevaluable, or unscored due to missing data. Of the 1,358 survivors participating in both SJLIFE and CCSS who completed the baseline CCSS survey, 595 did not complete surveys at T2 or T3, and 28 had missing data, leaving 735 participants for analyses (Supplementary Figure S1). This study was approved by the SJCRH Institutional Review Board, and all participants provided informed consent.

### Data Collection

Participants self-reported socio-demographics, symptoms, lifestyle behaviors, and health status via online or paper surveys during three time periods: 1994–2012 (T1), 2007–2013 (T2), and 2008–2015 (T3). Clinical data were obtained from clinical assessments conducted during each visit to SJLIFE through 2020, which included medical history review, physical exams, cognitive and functional evaluations, laboratory testing, and organ function evaluations.

### Symptom Measurement

A 37-item symptom survey was administered in both SJLIFE and CCSS, covering 10 domains: cardiac (3 items), respiratory (2 items), musculoskeletal (4 items), nausea (1 item), sensory (8 items), pain (4 items), fatigue (2 items), memory (1 item), anxiety (6 items), and depression (6 items) (Supplementary Table S1). Among 37 items, 19 were developed by CCSS investigators and 18 were adopted from the Brief Symptom Inventory-18.^20^ These items align with the Children’s Oncology Group Long-Term Follow-Up Guidelines (COG LTFU Guidelines) to assess treatment-related toxicities and have demonstrated sensitivity to treatment exposures.^7,8^ This symptom measure has been reported in a previous SJLIFE publication^11,12^ (see Supplementary Table S1 for the content and measurement properties). We used a checklist approach to classify each symptom domain as present if one or more items within that domain were endorsed by survivors.

### Classification of CHCs

CHCs were identified through review of electronic health records (EHRs) for medical history and clinical assessments during SJLIFE visits. The severity of 47 individual CHCs was graded using a modified version of the CTCAE, categorized as none, mild (grade 1), moderate (grade 2), severe/disabling (grade 3), or life-threatening (grade 4) (Supplementary Table S2).^21^ Individual CHCs were dichotomized as moderate/severe/life-threatening (grades 2–4) or absent/mild (none or grade 1). These 47 individual CHCs were classified into seven organ system-based groups: cardiac, vascular, respiratory, musculoskeletal, neurologic, endocrine, and reproductive. A given organ system was considered affected if any condition within that group was graded 2–4 in severity. Based on a previous study,^22^ all conditions were combined to classify total CHC burden by none/low (all CHCs grade 0 or 1), moderate (one or more grade 2 and/or one grade 3), high (two or more grade 3 or one grade 4), and very high (two or more grade 4 or two or more grade 3 and one grade 4).

### Socio-demographic and Clinical Information

Additional information was obtained from surveys, including age at evaluation, sex, race/ethnicity, educational attainment, marital status, and cigarette smoking status. Detailed cancer therapy data were abstracted from medical records, including cancer diagnosis, age at diagnosis, chemotherapeutic agents, regions of radiotherapy, and major surgical procedures.

### Statistical Analysis

Principal component analysis was conducted separately at T1, T2, and T3 to identify latent factors underlying 10 symptom domains. The number of symptom factors was determined using eigenvalues > 1 and factor loadings > 0.4. Latent profile analysis was conducted using standardized factor scores derived from principal component analysis to identify specific symptom clusters, representing subgroups of survivors with distinct symptom burden patterns at each time point. The optimal number of symptom clusters was determined by lower Bayesian Information Criterion (BIC), a significant Voung-Lo-Mendell-Rubin likelihood ratio test, and higher entropy (see the Results section for the labels of individual symptom clusters).

Changes in symptom clusters over time were classified as improved/persistently low symptom burden, persistently moderate symptom burden, or increased/persistently high symptom burden. Changes in CHCs over time were classified as either progression or non-progression (for classification methods, see Supplementary Table S3 for symptom cluster change, and Supplementary Table S4 for CHC progression). Modified Poisson regression was conducted to assess temporal associations across 3 models (Supplementary Figure S2): 1) T1 symptom clusters and progression of total CHC burden (i.e., increased severity or persistently high burden) from pre-T1 to post-T1; 2) changes in symptom clusters from T1-T2 and progression of total CHC burden from T1-T2 to post-T2; and 3) changes in symptom clusters from T2-T3 and progression of total CHC burden from T2-T3 to post-T3. Similar models were employed to evaluate temporal associations between symptom clusters (at T1 and changes in cluster membership) and progressive CHCs (i.e., new onset, persistence, or increased severity) within each organ system group. Models were adjusted for factors known to be associated with symptom presence and CHCs, including age at evaluation, sex, smoking status, and treatment exposures per the COG LTFU Guidelines^7,8^ (Supplementary Table S2). All analyses were performed using SAS v9.4 (SAS Institute, Cary, NC) and Mplus v8.2 (Muthen & Muthen, Los Angeles, CA), with statistical significance set at two-sided p-value < 0.05.

## RESULTS

### Characteristics of Participants

Of 735 survivors, approximately 50% were female; 90% were non-Hispanic White; 70% were treated for leukemia or lymphoma (Table 1). The mean (±SD) ages at T1, T2, and T3 survey completion were 27.0 (±5.1), 35.9 (±6.9), and 40.1 (±7.3) years, respectively. Compared with those who did not complete surveys at T2 or T3, survivors included in the analysis were older at cancer diagnosis, and more likely to have received radiotherapy (all p’s <0.05; Supplementary Table S5).

### Prevalence of Symptom Clusters and Cluster Change Over Time

**Figure 1:**
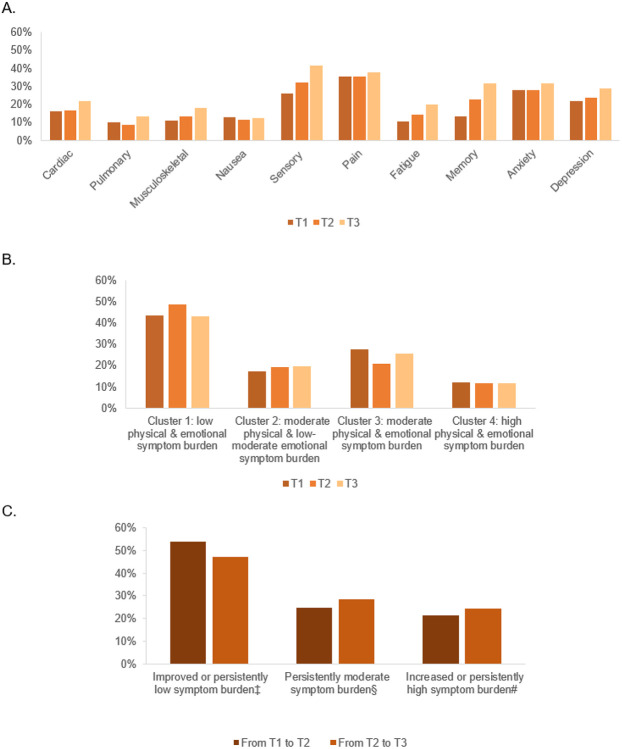
Prevalence of 10 symptom domains (Panel A) and symptom clusters^†^ (Panel B) at each time point and the change of symptom clusters over time (Panel C). ^#^ Increased or persistently high symptom burden (e.g., clusters 2 to 4, clusters 4 to 4) ^†^ See Supplementary Table S3 for the classification method of the change of symptom clusters ^‡^ Improved or persistently low symptom burden (e.g., clusters 4 to 2, clusters 1 to 1) ^§^ Persistently moderate symptom burden (e.g., clusters 2 to 2, clusters 3 to 3)

Principal component analysis identified two latent factors across 10 symptom domains at each time point, corresponding to physical-related and psychological-related factors. Based on this two-factor structure, latent profile analysis classified survivors into four consistent symptom clusters across time (Figure 1). The four clusters represented survivors with 4 symptom profiles: low physical and emotional symptoms (42.9-48.4% over time), moderate physical and low emotional symptoms (17.3-19.7%), moderate physical and emotional symptoms (20.8-27.3%), and high physical and emotional symptoms (11.6-12.0%). Survivors with changes in symptom clusters from T1 to T2 and from T2 to T3 were grouped into three categories: improved/persistently low symptom burden (T1 to T2: 53.9%, T2 to T3: 47.3%), persistently moderate symptom burden (T1 to T2: 24.8%, T2 to T3: 28.3%), and increased/persistently high symptom burden (T1 to T2: 21.4%, T2 to T3: 24.4%) (see Supplementary Table S3 for methods in classifying cluster changes).

### Prevalence and Progression of CHCs

**Figure 2:**
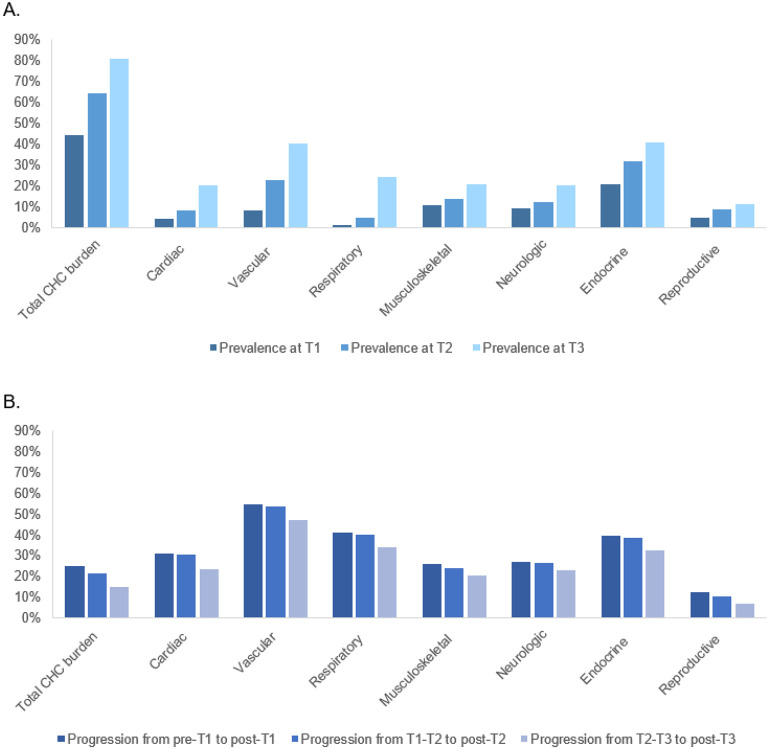
Prevalence of CHCs^†^ by time points (Panel A) and change over time (Panel B) at the total^‡^ and individual group^§^ levels. CHCs = Chronic health conditions † Individual 47 CHCs were graded and grouped by 7 CHC groups. Each of 47 CHCs was graded by modified CTCAE criteria as no conditions, Grade 1 (mild), Grade 2 (moderate), Grade 3 (severe/disabling) and Grade 4 (life-threatening). The highest grade of a specific CHC within a corresponding CHC group was chosen to represent the severity of a CHC group; Grades ≥2 represented the presence of a specific CHC group. ^‡^ Total CHC burden was classified as none/low, moderate, high, and very high. None/low category represented having any Grade 1 CHCs, moderate category for having ≥1 Grade 2 and/or 1 Grade 3 CHCs, high category for ≥2 Grade 3, or 1 Grade 4 and 1 Grade 3 CHCs, and very high category for ≥2 Grade 4 or ≥2 Grade 3 and 1 Grade 4 CHCs. Moderate, high or very high category represented the presence of total CHC burden.

The prevalence of total and grades 2-4 CHC burden increased over time across all seven groups (Figure 2). At least moderate total CHC burden was observed in approximately 44% of survivors at T1, 64% at T2, and 81% at T3. Endocrine conditions were the most common individual CHC group, affecting 21% of survivors at T1, 32% at T2, and 41% at T3. Progressive total CHC burden occurred in approximately 25% of survivors from pre-T1 to post-T1, 22% from T1-T2 to post-T2, and 15% from T2-T3 to post-T3. Vascular CHCs exhibited the highest progression rate among individual CHC groups.

### Temporal Associations of Symptom Clusters at T1 with Progression of CHCs (Model 1)

Compared to survivors with low physical and emotional symptoms at T1, those with high physical and emotional symptoms at T1 had a relative risk (RR) of 1.64 (95% CI 1.14-2.36) for progressive total CHC burden from pre-T1 to post-T1. Survivors with moderate physical and low emotional symptoms at T1 had an RR of 1.53 (95% CI 1.14-2.06) for progressive total CHC burden during the same period (Table 2). In contrast, treatment exposures were not significantly associated with the progression of total CHC burden (Supplementary Table S6).

By organ system groups, compared to low physical and emotional symptoms at T1, survivors with high physical and emotional symptoms at T1 had a significantly higher risk of progressive CHCs from pre-T1 to post-T1, particularly for neurologic (RR 2.53, 95% CI 1.81-3.53), musculoskeletal (RR 1.91, 95% CI 1.35-2.69), respiratory (RR 1.32, 95% CI 1.04-1.67), and vascular (RR 1.24, 95% CI 1.02-1.52) CHCs (Table 3). Survivors with moderate physical and low emotional symptoms at T1 had a 2.23-, 1.43-, and 1.29-fold increased risk of progressive neurologic, musculoskeletal, and respiratory CHCs, respectively (all p’s <0.05).

Specific chemotherapy or radiotherapy exposures were associated with progression of select CHC groups from pre-T1 to post-T1 (Supplementary Table S7). For example, anthracycline exposure was significantly associated with an increased risk of progressive cardiac CHCs (RR 1.49), while neck/chest irradiation was associated with progressive respiratory (RR 2.11) and endocrine (RR 2.16) CHCs (all p’s <0.05).

### Temporal Associations of Symptom Cluster Change from T1-T2 (Model 2) and from T2-T3 (Model 3) with Progression of CHCs

Survivors with increased or persistently high symptom burden between T1-T2 had a 1.63-fold higher risk of progressive total CHC burden (95% CI 1.18-2.26) from T1-T2 to post-T2, while those with a persistently moderate symptom burden had a 1.42-fold higher risk (95% CI 1.03-1.97), compared to those with improved or persistently low symptom burden (Table 2). Changes in symptom burden between T1-T2 were significantly associated with CHC progression from T1-T2 to post-T2 across CHC groups (Table 3). Specifically, survivors with increased/persistently high symptom burden had elevated risks of progressive neurologic (RR 2.23, 95% CI 1.69-2.93) and respiratory (RR 1.30, 95% CI 1.06-1.59) CHCs compared to those with improved/persistently low symptom burden between T1-T2. Additionally, survivors with persistently moderate symptom burden between T1-T2 had a 1.79-fold higher risk (95% CI 1.33-2.40) of progressive neurologic CHCs, compared to those with improved/persistently low symptom burden.

Survivors with increased/persistently high symptom burden between T2-T3 had a 2.28-fold (95% CI 1.51-3.45) higher risk of progressive total CHC burden from T2-T3 to post-T3, while those with persistently moderate symptom burden had a 1.66-fold (95% CI 1.07-2.59) higher risk (Table 2). By organ system groups, increased/persistently high symptom burden between T2-T3 was significantly associated with higher risks of progressive neurologic (RR 2.81, 95% CI 2.04-3.87) and respiratory (RR 1.33, 95% CI 1.06-1.68) CHCs from T2-T3 to post-T3. Additionally, survivors with persistently moderate symptom burden during the same period had a 1.74-fold (95% CI 1.21-2.50) higher risk of progressive neurologic CHCs compared to those with improved/persistently low symptom burden.

Treatment modalities were not significantly associated with progressive total CHC burden in Models 2 or 3 (Supplementary Table S6). However, certain chemotherapy and radiotherapy exposures were significantly associated with the progression of individual CHC groups (Supplementary Tables S8 and S9). For example, anthracycline exposure was associated with progressive cardiac CHCs post-T3 (RR 1.29), while neck/chest irradiation was associated with progressive respiratory CHCs (RR 2.30) and endocrine CHCs (RR 2.19) post-T3 (all p’s <0.05).

## DISCUSSION

Using 25 years of PRO survey data and clinically ascertained CTCAE-graded CHCs, we provide the first empirical evidence that symptom clusters predict future adverse medical events in childhood cancer survivors. High physical and emotional symptom burden at baseline, as well as increased or persistently high symptom burden over time, were strongly associated with progression of total and specific CHCs. These associations remained significant after adjusting for treatment, socio-demographic, and lifestyle factors.

The finding of a high symptom burden preceding CHC progression highlights a critical window for early detection and intervention. Recognizing these associations may enable timely clinical action to prevent complications and reduce healthcare costs.^23^ However, current survivorship surveillance often lacks standardized, routine symptom assessment, with cost-effective screening limited to high-risk survivors for specific conditions such as cardiomyopathy or subsequent neoplasms.^24^ While symptom tools are widely used during active cancer treatment,^25,26^ their integration into long-term survivorship care remains uncommon due to persistent implementation barriers.^27^ Recent advances in EHR-integrated symptom reporting have now made routine digital monitoring increasingly feasible.^28^ Our findings of temporal symptom-CHC associations suggest that incorporating regular symptom assessment between clinic visits could complement existing surveillance strategies, detect early signs of CHC progression, and support timely preventive care.

A growing body of evidence reinforces the clinical utility of symptom self-reporting. Observational studies across cancer and non-cancer populations have shown that self-reported symptoms can identify individuals in need of early medical evaluation.^15^ Recent randomized trials further demonstrate that embedding symptom tracking in care pathways reduces symptom burden among pediatric cancer patients.^29^ Building on symptom assessments recommended in the COG LTFU Guidelines,^7,8^ our findings support the use of sequential symptom surveys to identify survivors at elevated risk for specific CHCs. Routine symptom monitoring through EHRs may facilitate early CHC detection, improve QOL, and reduce long-term complications.^30^ Many symptoms and CHCs are also modifiable through lifestyle interventions, such as improving diet, increasing physical activity, promoting sleep, and reducing stress.^31,32^ Future research should assess the feasibility, scalability, and economic viability of systematic symptom surveillance in survivorship care, particularly in low-resource settings. For example, comparative studies could investigate whether real-time digital symptom monitoring (e.g., every 6 months) yields better health outcomes than usual care, thereby demonstrating the added value of proactive symptom tracking.^33^

The robust prognostic value of symptom burden for subsequent CHC development suggests that some survivors with moderate or high symptom burden may not yet have developed or been diagnosed with CHCs. When symptoms lack an identifiable structural or clinical pathology, they are often classified as “medically unexplained symptoms,” which may stem from cancer-related risk factors or other unknown reasons. These symptoms are linked to poorer QOL ^23^ and increased risk of depression,^34^ highlighting their importance in follow-up care. Using a symptom cluster approach provides a more comprehensive view of the patient’s experience by identifying patterns across multiple co-occurring symptoms, rather than examining isolated or unexplained complaints individually. Identifying symptom clusters associated with CHC progression (e.g., increased or persistently high symptom burden linked to neurologic CHCs) can inform early clinical interventions. For high-risk survivors, integrating palliative care may help address both explained and unexplained symptoms and improve late-effect management.^35^

The comparable prognostic value of baseline symptom burden and increasing symptom severity across most CHC groups suggests a shared symptom-based pattern in CHC progression. These findings provide a foundation for investigating the biological mechanisms linking specific symptoms to disease development. CHCs marked by co-occurring symptoms may share genetic risk loci or pathways.^36^ Symptom burden signatures could guide the development of individualized diagnostic tools and targeted therapies to better address co-occurring symptoms. Currently, no studies have explored the biological underpinnings of multiple co-occurring symptoms, highlighting a critical gap for future research.^13^ Treatment exposures likely precede both biological mechanisms and symptoms in the etiologic pathway, which occur before CHC diagnosis; however, this sequence remains untested and cannot be evaluated with the current data. To advance this line of inquiry, conceptual frameworks are needed to clarify the relationships among cancer treatment exposures, biological mechanisms, symptom clusters, and CHC progression for childhood cancer survivors.^13^

Interestingly, exposure to anthracycline agents and neck/chest radiotherapy, rather than baseline symptom burden or severity changes over time, was significantly associated with progressive cardiac conditions. The absence of a symptom-based association may refiect the use of broader measures of symptom burden rather than cardiac-specific symptoms. Previous studies have shown that early-stage cardiac conditions often present with symptoms such as chest pain and dyspnea,^37^ and that uncontrolled anxiety and depression are linked to adverse cardiac events in individuals with stable coronary artery disease or heart failure.^38,39^

This study has several limitations. First, analyses were limited to survivors who completed three symptom surveys. Those who had incomplete symptom survey data or died earlier were excluded, which limits the generalizability to the broader population of adult survivors of childhood cancer. Second, the sample was predominantly white and non-Hispanic, which further restricts the applicability of findings to diverse populations. Third, symptom data were collected only during long-term survivorship (i.e., a mean of 18 years post-diagnosis at T1), excluding symptoms experienced during cancer therapy or earlier phases of survivorship. A subsequent study of young (i.e., 8–18 years of age) survivors of childhood cancer found that 38% reported moderate to high symptom burden early in survivorship.^40^ Longitudinal symptom data from diagnosis through post-therapy and across the lifespan are needed to capture symptom evolution and its relationships with CHC progression. Future research should also determine the optimal timing, interval, and frequency for symptom assessment, ideally using systems that trigger alerts to healthcare teams when symptoms exceed predfiened clinical thresholds. Lastly, our findings do not establish the predictive validity of symptom data for subsequent adverse medical events. To support risk stratification and targeted intervention for cancer survivors, future research must evaluate the predictive performance of symptom assessments (e.g., accuracy, sensitivity, specificity, predictive values) using training and test datasets, along with standard assessment tools, such as the Patient-Reported Outcomes version of the Common Terminology Criteria for Adverse Events (PRO-CTCAE).

In conclusion, baseline symptom clusters and their changes over time independently link to subsequent CHC development in adult survivors of childhood cancer, beyond treatment exposures and traditional risk factors. Future research should focus on developing, validating, and implementing survivor-reported symptom assessments for risk prediction. Meanwhile, incorporating serial symptom assessments into risk-based follow-up care and clinical guidelines may help identify survivors who are at an elevated risk of late effects.

## Supplementary Material

This is a list of supplementary files associated with this preprint. Click to download.

• SUPPLEMENTARYMATERIALS.docx

## Figures and Tables

**Table 1:** Characteristics of study participants (N=735)

Characteristics	Mean (SD) or n (%)
Socio-demographic factors	
Mean age at symptom evaluation (in years)	
T1	27.0 (5.1)
T2	35.9 (6.9)
T3	40.1 (7.3)
Mean years since cancer diagnosis	
T1	17.7 (4.6)
T2	26.5 (6.5)
T3	30.7(6.9)
Sex	
Male	375 (51.0%)
Female	360 (49.0%)
Race/ethnicity	
White, non-Hispanic	660 (89.8%)
Other	75 (10.2%)
Educational attainment at T1	
Below high school, high school graduate or training after high school	502 (68.3%)
College graduate or postgraduate level	233 (31.7%)
Lifestyle factors	
Cigarette smoking at T1	
Never smoker	493 (67.4%)
Past smoker	105 (14.3%)
Current smoker	134 (18.3%)
Cigarette smoking at T2	
Never smoker	532 (73.0%)
Past smoker	72 (9.9%)
Current smoker	125 (17.2%)
Cancer diagnosis	
Leukemia	304 (41.4%)
Hodgkin lymphoma	156 (21.2%)
Non-Hodgkin lymphoma	64 (8.7%)
Osteosarcoma	50 (6.8%)
Wilms tumor	44 (6.0%)
Central nervous system tumor	32 (4.4%)
Neuroblastoma	30 (4.1%)
Soft tissue sarcoma/rhabdomyosarcoma	24 (3.3%)
Ewing sarcoma	23 (3.1%)
Other	8 (1.1%)
Chemotherapy	
Alkylating agents	491 (66.8%)
Anthracyclines	443 (60.3%)
Bleomycin	50 (6.8%)
Corticosteroids	411 (55.9%)
High-dose methotrexate	167 (22.7%)
Platinum	42 (5.7%)
Vincristine	565 (76.9%)
Radiotherapy	
Abdominal/pelvic irradiation	217 (29.5%)
Brain irradiation	302 (41.1%)
Neck/chest irradiation	265 (36.1%)
Total body irradiation	18 (2.5%)
**Bone marrow transplantation**	26 (3.5%)
**Major surgery (including limb-sparing surgery)**	427 (58.1%)

**Table 2:** Symptom clusters at T1 and cluster changes over time associated with progression of total CHC burden

Symptom clusters at T1 and cluster changes over time	Progression of total CHC burden
	RR (95% C.I.)^#^
Model 1: Symptom clusters at T1	Progression of total CHC burden post-T1
Low physical & emotional symptom burden	Reference
Moderate physical & low emotional symptom burden	1.53 (1.14, 2.06)**
Moderate physical & emotional symptom burden	0.94 (0.63, 1.40)
High physical & emotional symptom burden	1.64 (1.14, 2.36)**
Model 2: Change of symptom clusters from T1 to T2	Progression of total CHC burden from T1-T2 to post-T2
Improved or persistently low symptom burden^†^	Reference
Persistently moderate symptom burden^‡^	1.42 (1.03, 1.97)*
Increased or persistently high symptom burden^§^	1.63 (1.18, 2.26)**
Model 3: Change of symptom clusters from T2 to T3	Progression of total CHC burden from T2-T3 to post-T3
Improved or persistently low symptom burden^†^	Reference
Persistently moderate symptom burden^‡^	1.66 (1.07, 2.59)*
Increased or persistently high symptom burden^§^	2.28 (1.51, 3.45)***

CHCs = Chronic health conditions; RR = Relative risk

† Improved or persistently low symptom burden (e.g., clusters 4 to 2, clusters 1 to 1)

‡ Persistently moderate symptom burden (e.g., clusters 2 to 2, clusters 3 to 3)

§ Increased or persistently high symptom burden (e.g., clusters 2 to 4, clusters 4 to 4)

# Adjusted for age, sex, time since diagnosis, cigarette smoking, and treatment modalities listed in Supplementary Table S2

* p <0.05

** p<0.01

*** p<0.001

**Table 3:** Symptom clusters at T1 and cluster changes over time associated with progression of individual CHC groups

Symptom clusters at T1 and clusters change over time	Cardiac	Vascular	Respiratory	Musculoskeletal	Neurologic	Endocrine	Reproductive
	RR (95% C.I.)	RR (95% C.I.)	RR (95% C.I.)	RR (95% C.I.)	RR (95% C.I.)	RR (95% C.I.)	RR (95% C.I.)
Model 1: Symptom clusters at T1	Progression of individual CHC groups post-T1^†^						
Low physical & emotional symptom burden	Reference	Reference	Reference	Reference	Reference	Reference	Reference
Moderate physical & low emotional symptom burden	1.13 (0.88, 1.44)	1.12 (0.95, 1.32)	1.29 (1.07, 1.56)**	1.43 (1.05, 1.94)*	2.23 (1.65, 3.00)***	1.16 (0.95, 1.43)	1.32 (0.86, 2.04)
Moderate physical & emotional symptom burden	0.84 (0.60, 1.16)	1.38 (1.17, 1.62)***	0.99 (0.76, 1.30)	1.16 (0.80, 1.66)	1.29 (0.86, 1.92)	1.12 (0.87, 1.45)	0.96 (0.56, 1.65)
High physical & emotional symptom burden	0.96 (0.68, 1.35)	1.24 (1.02, 1.52)*	1.32 (1.04, 1.67)*	1.91 (1.35, 2.69)***	2.53 (1.81, 3.53)***	1.29 (0.99, 1.68)	1.51 (0.86, 2.66)
Model 2: Change of symptom clusters from T1 to T2	Progression of individual CHC groups from T1-T2 to post-T2^†^						
Improved or persistently low symptom burden^‡^	Reference	Reference	Reference	Reference	Reference	Reference	Reference
Persistently moderate symptom burden^§^	1.20 (0.93, 1.55)	1.21 (1.04, 1.41)*	1.30 (1.06, 1.60)*	0.99 (0.71, 1.39)	1.79 (1.33, 2.40)***	1.09 (0.88, 1.34)	1.52 (0.98, 2.36)
Increased or persistently high symptom burden^#^	1.16 (0.90, 1.50)	1.12 (0.94, 1.32)	1.30 (1.06, 1.59)*	1.28 (0.94, 1.74)	2.23 (1.69, 2.93)***	1.04 (0.82, 1.32)	1.17 (0.68, 2.03)
Model 3: Change of symptom clusters from T2 to T3	Progression of individual CHC groups from T2-T3 to post-T3^†^						
Improved or persistently low symptom burden^‡^	Reference	Reference	Reference	Reference	Reference	Reference	Reference
Persistently moderate symptom burden^§^	0.95 (0.69, 1.31)	1.10 (0.92, 1.32)	1.17 (0.92, 1.49)	1.49 (1.06, 2.10)*	1.74 (1.21, 2.50)**	1.28 (1.02, 1.62)*	2.41 (1.30, 4.45)**
Increased or persistently high symptom burden^#^	1.15 (0.85, 1.55)	1.18 (0.98, 1.42)	1.33 (1.06, 1.68)*	1.23 (0.85, 1.77)	2.81 (2.04, 3.87)***	1.30 (1.00, 1.68)*	1.80 (0.94, 3.45)

CHCs = Chronic health conditions; RR = Relative risk

† Adjusted for age, sex, time since diagnosis, cigarette smoking, and treatment modalities listed in Supplementary Table S2

‡ Improved or persistently low symptom burden (e.g., clusters 4 to 2, clusters 1 to 1)

§ Persistently moderate symptom burden (e.g., clusters 2 to 2, clusters 3 to 3)

# Increased or persistently high symptom burden (e.g., clusters 2 to 4, clusters 4 to 4)

* p <0.05

** p<0.01

*** p<0.001
